# Efficacy of Probiotics in Improving Motor Function and Alleviating Constipation in Parkinson’s Disease: A Randomized Controlled Trial

**DOI:** 10.5812/ijpr-137840

**Published:** 2023-09-01

**Authors:** Nasibeh Ghalandari, Farhad Assarzadegan, Seyed Amir Hassan Habibi, Hadi Esmaily, Habib Malekpour

**Affiliations:** 1 Department of Clinical Pharmacy, School of Pharmacy, Shahid Beheshti University of Medical Sciences, Tehran, Iran; 2 Department of Neurology, School of Medicine, Shahid Beheshti University of Medical Sciences, Tehran, Iran; 3 Department of Gastroenterology and Liver Diseases, School of Medicine, Iran University of Medical Sciences, Tehran, Iran

**Keywords:** Parkinson’s Disease, Bristol Stool Scale, Constipation, Probiotics, UPDRS

## Abstract

**Background:**

Parkinson’s disease (PD) is one of the common neurodegenerative diseases, and there has been an increasing interest in the potential role of intestinal dysbiosis in its pathogenesis and related gastrointestinal complications such as constipation.

**Objectives:**

This study aims to evaluate the effects of multi-strain probiotics on constipation and motor function in PD patients.

**Methods:**

This study was a blinded, randomized controlled trial (RCT) that involved 27 PD patients who were diagnosed with constipation according to the ROME IV criteria for functional constipation. The primary outcome measured before and after the intervention in both the placebo and probiotic groups was the frequency of defecation. Secondary outcomes evaluated were laxative use, sense of complete evacuation, Bristol Stool Scale for consistency, and Unified Parkinson’s Disease Rating Scale (UPDRS) scale. The study lasted for eight weeks. Both groups also were educated about lifestyle modification.

**Results:**

Of 30 included patients (15 in each group), 13 were women, and 17 were men. Three patients dropped out of the study. Between-group analysis showed that the frequency of bowel movements significantly increased in the probiotic group 4 [3 - 5] in comparison with 2 [2 - 3] in placebo (P = 0.02). Stool consistency also improved in the probiotic group (P = 0.04). However, there were no significant differences in other outcomes. The within-group analysis showed improvement in stool consistency in both probiotics and placebo groups (P = 0.01 and P = 0.007, respectively), while stool frequency and sense of complete evacuation significantly improved only in the probiotic group (P < 0.05).

**Conclusions:**

This study demonstrated that multi-strain probiotics could improve frequency, consistency, and sense of complete evacuation in PD patients, while there was no significant effect on motor functions in 8 weeks. It is suggested that additional studies be conducted on longer-term effects.

## 1. Background

This paper discusses Parkinson’s disease (PD), a neuromuscular disorder prevalent in elderly patients (1). One of the non-motor complications that PD patients often face is constipation, which can be troublesome (2). Laxatives, such as macrogol and lubiprostone, have improved stool frequency and consistency in PD patients (3, 4).

Intestinal dysbiosis, an imbalance of gut bacteria, has been linked to neurological diseases (5). There is also a theory that the brain-gut axis plays a role in the pathogenesis of PD (4). The metabolites produced by gut bacteria can have harmful or beneficial effects on the human body’s immune, neural, and hormonal systems (5).

Probiotics are useful microorganisms that can alter gastrointestinal and immune responses in different diseases, such as irritable bowel syndrome, traveler’s diarrhea, and inflammatory bowel disease (6). It has been proposed that probiotics can modulate host immunity by affecting the intestinal barrier, which can lead to a decrease in immune response (7).

Evidence has shown that a decline in the population of *Lactobacillus* and *Bifidobacterium* in adults can cause constipation (8). Therefore, it is suggested that multi-strain probiotics, consisting of a combination of these two genera, be used to treat constipation. It has also been demonstrated that *Lactobacillus* and *Bifidobacterium* can accelerate small intestine transit, likely due to the release of serotonin (5-HT) and its effects on motility (9).

A recent meta-analysis (1) of numerous studies (2-4, 10, 11) has shown that probiotics can increase bowel movement frequency in PD patients with constipation. In this review, different specific mechanisms of Parkinson’s constipation, such as a-synuclein aggregation, dysbiosis, and motility dysfunctions, have been discussed. Additionally, some studies have found significant reductions in metabolic and inflammatory markers (12, 13). On motor function, research has been inconclusive, as two articles have investigated the effects of probiotic supplementation on motor symptoms, with one reporting a reduction in the Unified Parkinson’s Disease Rating Scale (UPDRS) scale (13) and the other reporting no significant changes (3).

## 2. Objectives

This study aimed to evaluate the effectiveness of probiotics on various aspects of constipation, such as frequency, laxative use, sense of complete evacuation, stool consistency, and UPDRS scale.

## 3. Methods

### 3.1. Design

Between September 2022 and January 2023, a triple-blind, parallel, randomized, and controlled trial was conducted. This study was in accordance with Consolidated Standards of Reporting Trials (CONSORT) guidelines.

### 3.2. Ethical Consideration

This trial was performed in accordance with the Declaration of Helsinki, and was registered with the Ethics Committee of the Faculty of Pharmacy at the Shahid Beheshti University of Tehran with the code IR.SBMU.PHARMACY.REC.1401.011 on March 15, 2022. Additionally, the study was registered on the Iranian Registry of Clinical Trials (IRCT) website, a primary register established by the World Health Organization (WHO). The registration number for the study was IRCT20170608034390N11, and it was completed on May 14, 2022.

### 3.3. Objectives and Interventions

This study aimed to investigate the effects of probiotic consumption on constipation in PD patients, as determined by the frequency of bowel movements. Secondary outcomes were also evaluated, including stool consistency, sense of complete evacuation, reduction in laxative use, and motor function. The intervention group received Comflor® (Fara Daroo Fanavar Mehr Co) capsules containing a total of 4.5 × 10^11^ CFU of *Lactobacillus plantarum*, *Lactobacillus casei*, *Lactobacillus acidophilus*, *Lactobacillus bulgaricus*, *Bifidobacterium infantis*, *Bifidobacterium longum*, *Bifidobacterium breve*, and *Streptococcus thermophilus* (each genus accounting for 1.5 × 10^11^ CFU) without a prebiotic component. The control group received the same company’s placebo capsules containing maltodextrin and starch.

### 3.4. Sample Size

In 2020, Ibrahim et al. (3) reported that patients receiving probiotics had an average of 4.18 ± 1.44 defecations per week, while patients receiving placebo had an average of 2.81 ± 1.06 defecations per week. The effect size was calculated to be 1.08 using this data. To conduct a study with the same parameters, an independent sample t-test was used with an alpha error of 0.05 and a power of 80%. Based on these calculations, a sample size of 24 in total was needed, and accounting for a 20% withdrawal rate due to the outpatient nature of the study, a total of 30 patients, 15 in each group, was determined.

### 3.5. Randomization and Blinding

To randomize subjects, the study employed the block randomization method. Specifically, the Sealed Envelope online software was used to generate 5 blocks, each consisting of 6 patients, forming 15 patients in each group. The groups were labeled as either Placebo or Probiotics, with 15 generated codes assigned to each group. To ensure blinding, the patients, researchers, and analysts remained blinded throughout the trial until the completion of the analysis. This was achieved by providing identical opaque capsules with identical packaging.

### 3.6. Eligibility

This study included 60 or older participants who presented to the neurology clinic of Imam Hossein Hospital in Tehran. Inclusion criteria were a diagnosis of idiopathic PD, providing written consent, and a history of constipation based on the Rome IV criteria for functional constipation. Participants with known allergies to probiotics, active infections, taking antibiotics, age under 60, using other probiotics, regularly taking laxatives, immunocompromised, using traditional medicine products to relieve constipation, or taking drugs with a higher than 10% risk of constipation as an adverse effect in their drug profile (except for Food and Drug Administration (FDA)-approved drugs in the treatment of PD) were excluded.

### 3.7. Requirement

All participants were administered one capsule per day for eight weeks during the study period. The patient’s drug history and supplement use were asked and recorded in case forms complying with good clinical practice principles. Follow-up assessments were conducted after the eighth week to evaluate any potential side effects as the end point of the study. The probiotic capsules were supplied for 8 weeks, and patients were instructed to inform the researchers if needed. Patients were given track diaries to record weekly data on bowel movements, laxative use, stool consistency, and the feeling of complete evacuation.

To minimize the impact of diverse lifestyles and dietary habits, every participant received a patient education pamphlet, nutritional training, and non-pharmacological interventions.

### 3.8. Outcome Measurement

Participants were given a tracking diary to assess the intervention’s effectiveness on constipation. Meanwhile, the improvement in motor function was evaluated using the UPDRS. The UPDRS is a questionnaire that was revised in 2007 and is composed of four domains: Part I, which assesses non-motor experiences of daily living; Part II, which evaluates motor experiences of daily living; Part III, which measures motor examination; and Part IV, which assesses motor complications (14). The validated Persian version of the second domain was acquired, and the third domain was translated for use in the study.

### 3.9. Adherence Assessment

To assess the adherence of the participants, the researchers utilized two methods. Firstly, the researchers called each enrolled patient every two weeks. Secondly, the pill count method was used to check medication adherence.

### 3.10. Statistical Analysis

The statistical analysis was carried out using the IBM Statistical Package for the Social Sciences (SPSS) version 23 (SPSS Inc., Chicago, IL, USA). Mean ± standard deviation was used to present parametric results. The normality of data distribution was assessed using the Shapiro-Wilk Kolmogorov test. Within-group analysis was used to compare data before and after treatment utilizing the paired sample *t*-test for variables with normal distribution such as UPDRS II and III, and the Related-Samples Wilcoxon signed rank test was utilized for the parameter of weekly bowel movements. Independent *t*-test analysis was used for between-group analysis in parametric variables. In contrast, the Mann-Whitney U ranking test was used for non-parametric variables such as weekly bowel movements and pill counting. The chi-square test was used for qualitative data, such as examining the distribution of gender and frequency of background disorders in the two groups. The significance level was less than 0.05 (P < 0.05).

## 4. Results

**Figure 1.**
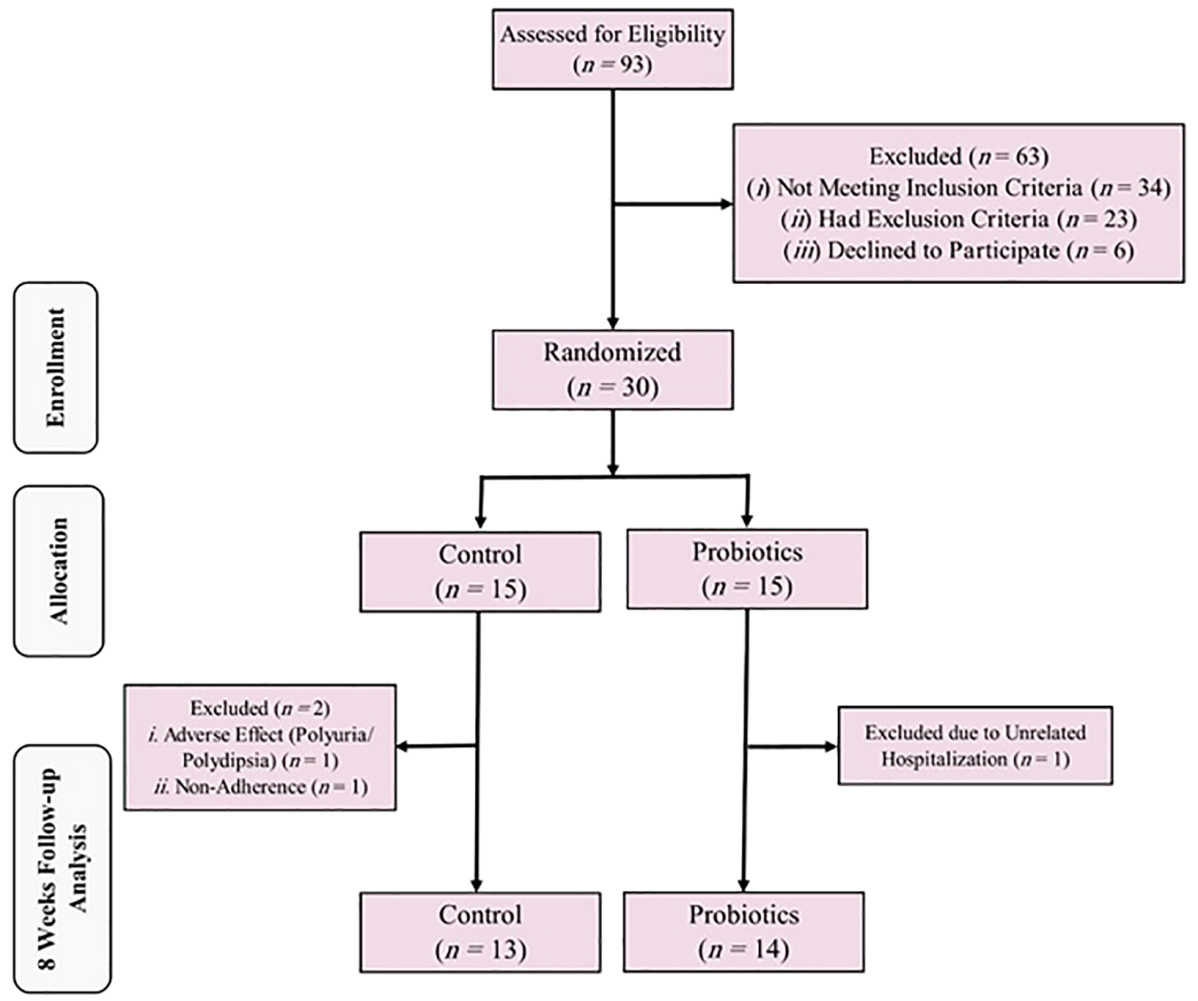
The Study’s Consolidated Standards for Reporting Trials (CONSORT) flow chart

Among the 93 patients assessed initially, only 30 (13 women and 17 men) met the eligibility criteria and were enrolled in the study, as shown in Figure 1. 

All enrolled patients were on levodopa and one DOPA decarboxylase, either benserazide or carbidopa; all drugs used to control their motor symptoms are tabulated in Table 1. One patient from the Probiotics group dropped out due to hospitalization for pyelonephritis unrelated to the trial. Additionally, two Placebo group members dropped out due to issues with adherence and adverse effects, respectively. Demographic and baseline characteristics were not significantly different among the patients. Table 1 provides a more detailed overview of the baseline parameters.

**Table 1.** Baseline Parameters and Characteristics ^a, b^

Baseline Parameters and Characteristics	Placebo (n = 13)	Probiotics (n = 14)	P Value
**Age (y)**	68.54 ± 6.92	68.07 ± 6.68	0.86
**Length of diagnosis (y)**	6.00 ± 3.63	4.43 ± 2.38	0.19
**Dosage of levodopa/day (mg)**	646.15 ± 145.00	607.14 ± 304.99	0.68
**Comorbidities**			0.43
None	3	6	
Diabetes	5	2	
Cardiovascular	5	7	
Depression	5	2	
Chronic kidney disease	1	0	
Rheumatoid arthritis	0	1	
Benign prostate hypertrophy	1	0	
Hypothyroidism	0	1	
**Gender**			0.86
Female	6	6	
Male	7	8	
**Smoking**	0	1	0.34
**Medications used in addition to levodopa**			0.84
Pramipexole	4	3	
Apomorphine	1	0	
Amantadine	1	2	
Trihexyphenidyl/biperiden	0	0	
Hohn-Yahr Staging*			0.12
0	0	1	
1	0	3	
2	2	1	
3	6	3	
4	5	3	
5	0	3	

^a^ Values are expressed as mean ± standard deviation or n.

^b^ Stage 0, no movement symptoms; stage 1.0, unilateral involvement only.; stage 2.0, bilateral involvement without impairment of balance.; stage 3.0, mild to moderate bilateral involvement, with some postural instability, but still physically independent.; stage 4.0, severe disability, but the patient can still walk and stand unassisted.; stage 5.0, wheelchair-bound or bedridden unless aided

The medication adherence rate, determined by pill count and personal query, was 93% in the probiotics group compared to 90% in the placebo group (P = 0.39). Additionally, the consistency based on the Bristol Stool Scale at the study endpoint showed a significant improvement in both groups (P = 0.04), as shown in Table 2. However, there were no statistically significant differences between the two groups regarding other gastrointestinal outcomes, such as the number of defecations with a sense of complete evacuation per week or the use of laxatives, as displayed in Table 3. 

**Table 2.** Results of Stool Consistency

Bristol Stool Scale	Placebo	Probiotics	Between-Group Analysis P Value
**Baseline**			0.45
Severe constipation	4	6	
Constipation	9	7	
Normal	0	1	
**8th Week**			0.04
Severe constipation	5	0	
Constipation	6	11	
Normal (type 3)	2	2	
Normal (type 4)	0	1	
Within-group analysis P value	0.01	0.007	

**Table 3.** Primary and Secondary Outcome Results

Outcome Results	Placebo	Probiotics	Between-Group Analysis P Value
**Defecation per week (n)**			
Baseline	3 [2 - 3]	3 [2 - 3]	0.94
After eight weeks	2 [2 - 3]	4 [3 - 5]	0.02
Within-group analysis P value	0.417	0.001	
**Times of defecation with a sense of complete evacuation per week**			
Baseline	2.1 ± 1.5	2.1 ± 1.7	0.91
After eight weeks	2.3 ± 1.2	3.1 ± 2.2	0.27
Within-group analysis P value	0.53	0.03	
**Times needed to use laxative per week ** ^**a**^			
Baseline	2.3 ± 2.2	2.5 ± 2.3	0.74
After eight weeks	2.5 ± 1.6	1.6 ± 1.2	0.11
Within-group analysis P value	0.71	0.07	
**UPDRS II**			
Baseline	23.1 ± 5.6	21.8 ± 12.5	0.33
After eight weeks	22.8 ± 6.7	21.3 ± 13.5	0.71
Within-group analysis P value	0.77	0.40	
**UPDRS III**			
Baseline	46.8 ± 19.8	50.2 ± 27	0.93
After eight weeks	48.2 ± 20.5	50.3 ± 27.3	0.83
Within-group analysis P value	0.29	0.96	

Abbreviation: UPDRS, Unified Parkinson’s Disease Rating Scale.

^a^ The number of times of laxatives use per week

The probiotics group exhibited a significant (47%) improvement in the number of defecations with a sense of complete evacuation per week. In contrast, the placebo group only showed a 9% improvement, as demonstrated in Table 3. Although there was a reduction in the need for laxatives, this difference did not reach statistical significance. Additionally, both groups showed significant improvements in consistency, as assessed by the Bristol stool exam, as displayed in Table 2. 

The effect size of this study was calculated based on the primary outcome of defecation frequency, resulting in a value of 1.12. The power of the study showed a result of 81.7 percent.

Regarding adverse effects, two male participants in the placebo group reported polydipsia and polyuria during the study. One of these patients had a history of CKD, and the other had BPH; the same outcomes were repeated when they were rechallenged. Based on the estimated Naranjo scale, the adverse effects were classified as possible ADR with a score of 3. One of the participants dropped out of the study due to these complications.

## 5. Discussion

Constipation is common in PD patients and can significantly impact their quality of life. While some interventions have been used to treat constipation in PD patients, more evidence is still needed due to a lack of response or adverse effects. A meta-analysis of three randomized controlled trials (RCTs) found that probiotics had a significant effect on the frequency of bowel movements per week, with a standardized mean difference (SMD) of 0.92 (P < 0.001) (1). This suggests probiotics may affect all proposed mechanisms of PD patients’ constipation pathophysiology. Additionally, many GI symptoms appear years before motor symptoms in PD (15), indicating that GI symptoms may respond to treatment before motor complications. Another meta-analysis found that multi-strain probiotics effectively improved stool frequency and consistency (16).

Different species of probiotics have varying effects. For example, one study found that *Bifidobacterium subtilis* and *Bifidobacterium lactis* had a trivial effect on metabolic or cholesterol profile but increased deconjugated bile acids plasma concentration (17). Another study found that *Lactobacillus casei rhamnosus* could control respiratory infections (18). Liang et al. suggested that a proper combination of *Lactobacillus* and *Bifidobacterium* had pronounced effects compared with single genus or high-dosage consumption in patients with irritable bowel syndrome (19). This indicates that there may be a synergistic or additive effect when they are used in combination. To our knowledge, no study has been conducted on human (20) subjects to evaluate the effect of different probiotics on neuropsychiatric disorders. This could also support the hypothesis that there is a certain “optimal combination” for each disease.

Another area that requires more attention is the different clinical effects of prebiotics, probiotics, synbiotics, and a combination of probiotics and prebiotics in various diseases. This may assist in identifying the responsible products for different mechanisms, although the clinical differences might not be statistically significant. For instance, a study conducted on rats fed on a high-fat, low-fiber diet compared synbiotic supplementation of Lactobacillus, and Bifidobacterium, combined with 10% fructooligosaccharides (FOS) to FOS supplementation, which resulted in suppressed intestinal and systemic inflammation, and the effects were comparable (21).

The stool consistency of patients with functional constipation based on ROME IV criteria was improved after consuming probiotics for four weeks, as evaluated by the Bristol stool exam, according to an RCT (22). This study found that the effect persisted after discontinuing the probiotics, especially when using *L. plantarum*. In our study, we observed similar results: Probiotics can improve stool consistency based on the Bristol Stool Scale in PD patients with constipation. One possible mechanism for this effect is an increase in the fecal amount of short-chain fatty acids, which can control the growth of harmful microorganisms and slightly soften the stool (23).

To reduce bias due to different lifestyles, our study was the first to educate patients and evaluate the intervention’s impact on this condition. Kang et al. (24) conducted a study on patients with IBS and found that lifestyle modifications significantly increased the number of patients with type 3, 4, or 5 on the Bristol Stool Scale (P < 0.05).

Our study also supported the hypothesis that educating PD patients with constipation can improve stool consistency (P = 0.01). This finding suggests that lifestyle modifications may be more effective in improving stool consistency, which can be further enhanced by using probiotics, as demonstrated in our study (P = 0.007).

**Figure 2.**
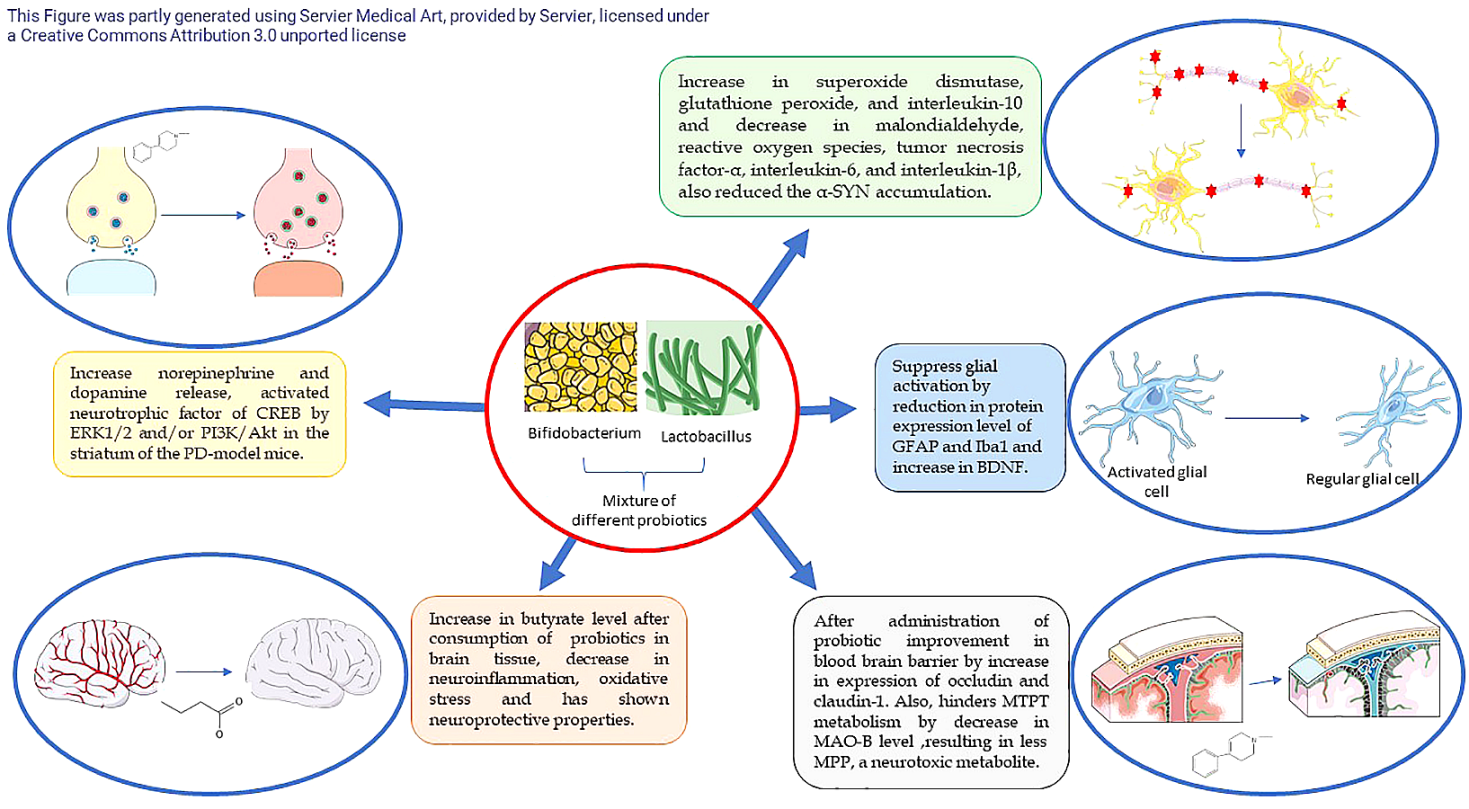
Different mechanisms of different probiotics strains on Parkinson’s disease (PD) (20, 25-28). CREB, cAMP-response element binding protein; ERK, extracellular signal-regulated kinase; GFAP, glial fibrillary acidic protein; Iba1, ionized calcium-binding adapter molecule1; MAO, monoamine oxidase; MPP, 1-methyl-4-phenylpyridinium; MPTP, 1-methyl-4-phenyl-1,2,3,6-tetrahydropyridinm; PI3K/Akt, phosphatidylinositol 3‑kinase/protein kinase B

Figure 2 illustrates the distinct mechanisms of various probiotic strains on PD.

Based on the reported effect size and power, it appears that the probiotic intervention in this study may benefit PD patients. However, the study did not find any significant difference between the probiotic and placebo groups regarding UPDRS scores. Previous research in this area has also produced conflicting results. We observed a difference between Tamtaji et al.’s (13) study and Ibrahim et al.’s (3) study. In Tamtaji et al.’s study, the patients’ intervention was labeled as synbiotic by the manufacturer (13). This labeling may have impacted the results, and a different outcome might have been observed if only probiotics had been administered. Additionally, in Ibrahim et al.’s study, the researchers scored and analyzed two different domains of UPDRS separately, while Tamtaji et al. analyzed the entire score (13). Since UPDRS includes four distinct domains, namely mood, daily life, motor action, and complication of therapy, it can be tentatively assumed that probiotics may have a more positive effect on mood and treatment complications. However, further research in this area is necessary to provide a more comprehensive understanding of the potential benefits of probiotics.

In contrast, Tamtaji et al. administered a probiotic combination of *Lactobacillus acidophilus*, *Lactobacillus rueteri*, *Bifidobacterium bifidum*, and *Lactobacillus fermentum* at a dose of 8 × 10^9^ CFU for 12 weeks, while Ibrahim et al. administered a combination of *L. casei*, *L. acidophilus*, *Lactobacillus lactis*, *B. infantis*, and *B. longum* at a dose of 30 × 10^9^ CFU for eight weeks (13). This study’s sample size was calculated based on Ibrahim et al.’s study, and there were many similarities between these two studies, such as length, CFU count, and probiotic genera (3). Thus, similar results are not unexpected.

As mentioned in the introduction, the gut-brain axis plays a newly discovered role in the pathogenesis of PD. This has been proposed to be a two-way route, and motor-symptom-preceding non-motor dysfunction is stated as evidence for this hypothesis (29). Also, altered permeability of enteric membrane in PD patients has been proven that in PD patients, and this significantly correlates with intestinal expression of α-syn, enteral *Escherichia coli* count, and levels of lipopolysaccharide-binding protein in serum. Still, the clinical importance of this finding remains to be studied (30). This theory indicates the possible importance of probiotics in managing Parkinson’s patients without motor symptoms.

However, it is important to note that the primary focus of our study was not on the impact of probiotics on improving UPDRS in PD patients and that neurological improvement is a complex issue that involves multiple factors. Before conducting our study, we hypothesized that probiotics may not significantly alleviate symptoms of PD and that other pharmacological interventions may be necessary to control the progression of the disease. Therefore, it is recommended that future studies investigate the use of different probiotic species on different aspects of UPDRS, with varying durations of treatment, to determine whether probiotics may offer greater benefits to patients with PD than what has already been suggested by previous research.

### 5.1. Limitations

The study encountered several challenges due to the COVID-19 pandemic. Additionally, the length of probiotic treatment was relatively brief, and the number of participants was limited.

### 5.2. Conclusions

Based on the findings of this study, the concurrent use of Comflor^®^ and lifestyle modification can effectively improve constipation in PD patients. This approach was observed to enhance bowel movement frequency, consistency, and a sense of complete evacuation and proved to be more effective than lifestyle modification alone. However, the study did not demonstrate significant differences in motor function based on UPDRS scores. Furthermore, patient evaluations indicated that probiotic consumption was well-tolerated.

## Clinical Trial Registration Code:

IRCT20170608034390N11.
